# An Overview of Prosopagnosia as a Symptom of Migraine: A Literature Review

**DOI:** 10.1007/s11916-025-01363-6

**Published:** 2025-02-19

**Authors:** Sidney Ley

**Affiliations:** Department of Biological Sciences, College of Arts and Sciences, Bowling Green State University, 704 Ridge St, Bowling Green, OH 43403 USA

**Keywords:** Migraine, Prosopagnosia, Migraine aura, Cortical dysfunction, Occipitotemporal cortex

## Abstract

**Purpose of Review:**

Prosopagnosia is a neurological phenotype, characterized by the inability to recognize faces, typically resulting from damage or dysfunction in specific brain regions such as the fusiform gyrus. In contrast, migraine is a disease process, a complex neurological disorder with a range of symptoms including severe headache and visual disturbances.

**Recent Findings:**

The brain regions involved in migraine and prosopagnosia are located in close proximity to each other, and perhaps as an unsurprising yet rarely reported result of this, there have been several cases of migraineurs, the majority presenting with aura, who manifested prosopagnosia as a symptom during an attack. While rarely reported, the fact that prosopagnosia can occasionally manifest during migraine episodes, particularly during the aura phase, emphasizes the importance of exploring the cortical processes involved in both conditions.

**Summary:**

This review discusses migraine and prosopagnosia in the context of comorbidity, explores and summarizes current and key historical knowledge on the reported occurrences of prosopagnosia manifesting as a symptom of migraine, and emphasizes the importance of reporting this phenomenon.

## Introduction

Migraine, a prevalent and often debilitating neurological disorder, and prosopagnosia, a rare and selective form of visual agnosia, seem unrelated at face value. However, case studies report that prosopagnosia can manifest as a symptom during migraine attacks, particularly during the aura phase. This overlap, though rarely reported, may provide crucial insights into the cortical processes underlying both conditions. Exploring the intersection of these two phenomena highlights an unusual but clinically significant aspect of migraine pathology, as migraine research remains in its infancy. This review aims to discuss the brain regions involved in migraine and prosopagnosia and summarize the reported case studies featuring prosopagnosia as a symptom of migraine.

### Mystery of the Migraine

Migraine is a common and often debilitating neurological condition characterized by severe headaches that can last from four to 72 h [1, 2]. Migraine may present with or without aura; migraine aura refers to a transient neurological symptom that coincides with or precedes the headache phase [3]. Research suggests that cortical dysfunction, including hyperexcitability and aberrant signaling [4], plays a key role in migraine pathology [5–9]. Migraine aura often involves transient disturbances such as dysphasia, impaired memory recall, and occasionally impaired facial recognition [9]. The visual cortex, particularly within the occipital lobe, is implicated in these disturbances [5, 10], with cortical spreading depression (CSD) now proposed as the cause of aura symptoms [11–13]. Notably, increased activity in the fusiform gyrus during migraine has also been reported [14], which may explain instances where migraine aura includes prosopagnosia.

### Problems with Prosopagnosia

Under normal circumstances, facial recognition is driven effortlessly by the facial recognition networks within the brain, the main driver of which is the fusiform gyrus [15], but also include the highly specialized fusiform face area within the fusiform gyrus [16]. Prosopagnosic patients, however, have dysfunction or physical damage to one or more of the structures within the brain devoted to facial recognition, most commonly the right fusiform gyrus [17, 18]. The result of this dysfunction or damage is selective visual agnosia that prevents easy facial recognition [19]. Prosopagnosia can either be developmental or acquired, with the latter often resulting from incidents that leave physical lesions, such as stroke, or physical trauma [20, 18, 21]. Acquired prosopagnosia typically involves damage to the right fusiform gyrus [18], but other areas within the facial recognition network have also been implicated [20, 22, 23, 16]. The proximity of these brain regions to those affected during migraine aura suggests a potential overlap that could explain the manifestation of prosopagnosia as a symptom during migraine attacks.

## Case Studies

**Fig. 1:**
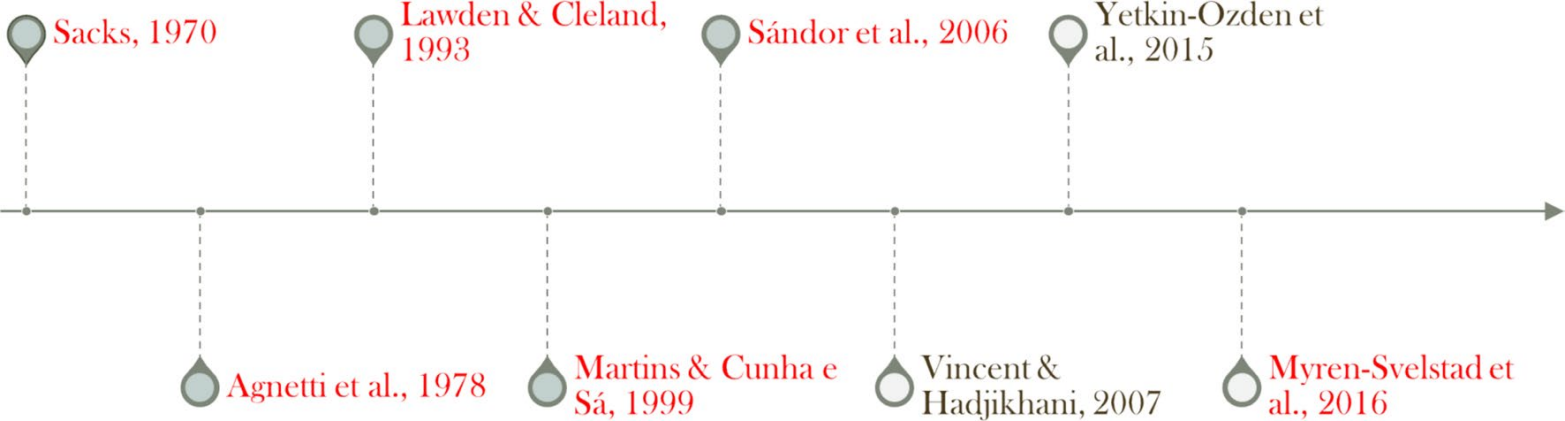
Timeline of migraine-prosopagnosia case reports and studies. The red citations are single-patient case studies, while the black citations are larger studies with multiple research participants

The proximity of brain regions implicated in both migraine and prosopagnosia suggests an overlap in cortical networks, particularly during the aura phase of migraine. The following section summarizes reports where prosopagnosia manifested as a symptom during migraine attacks, which provide insight on this rare yet clinically significant occurrence. Due to their rarity and symptom relevance, the notable cases involving prosopagnosia and migraine, e.g., all published cases that could be found, are summarized here in chronological order (Fig. 1).

The first formally mentioned occurrence of this phenomenon appears in the 1970 book *Migraine*, by Oliver Sacks, where he first describes his observance of the common loss of “synthetic” perception during migraine, e.g., prosopagnosia, during migraine attacks. A recount of English physician Edward Living is depicted here undergoing partial loss of facial recognition during scotoma with migraine, as well as a case of a 75-year-old-woman who, during her aura phase in a small sample of migraine attacks, experienced autotopagnosia, object agnosia, acoustic agnosia, and prosopagnosia [24]. A quality of the aura phase appears to make it more likely for prosopagnosia to occur during migraine attacks; in acquired prosopagnosia, the lesion location is frequently on the fusiform or lingular gyrus, within the right inferior or medial occipitotemporal cortex [18, 25]. A second mention of this phenomenon appears soon after in 1978, when Agnetti and colleagues published a case report on ictal prosopagnosia, and therein mentions that it is well-known in the clinical setting to encounter prosopagnosia as a paroxysmal symptom of both migraine and epilepsy [26]. For the third occurrence, in 1993 Lawden & Cleland report a case of a 49-year-old-woman, described as having transient neurological dysfunction during her migraine attack, the symptoms and comorbidities of which included prosopagnosia, topographic disorientation, and achromatopsia. Her migraine symptoms continued until pharmacological intervention, at which point the other symptoms resolved as well [27]. The fourth mention of this phenomenon in literature occurred in 1999, where Martins and colleagues describes a case of a 28-year-old female migraine patient who suffered from a complex right hemispheric dysfunction during her migraine attack, right at the aura phase. Her symptoms included left hemianopia, left-sided paresthesias, and then a loss of topographic and procedural memory, followed by prosopagnosia [28]. Considering its adjacent proximity to the visual cortices, damage to the visual cortex area may affect or clinically progress to the occipitotemporal cortex over some time, resulting in aura symptoms transitioning from visual field restrictions to prosopagnosia. In support of this, a fifth case reported by Sandor and colleagues in 2006 recounted a 58-year-old left-handed man periodically and repeatedly experiencing prosopagnosia and visual disturbances with his migraine, as well as transient global amnesia [29]. This particular case is also of note as it breaks the convention of only right-handed women experiencing prosopagnosia with their migraine attacks.

In 2007, the largest study to date that explored facial recognition, memory, and complex visual and color perception deficits among migraine patients discovered that seven of their 143 participants had prosopagnosia as a symptom of, or irrespective of, their migraines [30]. Of these seven, four (three females and one male) reported prosopagnosia occurring before the onset of a migraine attack, while three (two females and one male) reported having prosopagnosia on a daily basis. Of these, two of the females’ migraines presented without aura, breaking the previously established pattern of only migraine patients with aura experiencing prosopagnosia with their migraines. Other neurological symptoms were also reported in this study, including apraxia, anesthesia, weakness, and alien hand syndrome at high prevenances; data from this study, and others like it, imply that neurological symptoms other than head pain and aura may be vastly underestimated in migraine patients [31, 30]. Many instances of bizarre perceptions of migraine may go unreported, as patients may not find them relevant to mention to their care provider or fail to mention them out of fear of seeming “crazy” [32]. Interestingly, three of the prosopagnosic patients (two female, one male) in this study reported dyschromatopsia related to their migraines as well. While the visual cortex is implicated in many visual abnormalities seen in migraine, the prosopagnosia seen in this case implies the involvement of the fusiform gyrus or the inferior occipital cortex. Other areas, such as the anterior temporal cortex, are also implicated in prosopagnosia, but given the comorbidity of dyschromatopsia, which involves the V8 area, which lies very close to the fusiform gyrus, fusiform gyrus dysfunction is likely implicit here [30].

The second-largest study to date exploring facial recognition in migraineurs occurred in 2014, with 74 participants in the migraine group (61 women and 13 men) [33]. These patients underwent the Benton face recognition test; patients with migraine scored significantly lower than the controls, and surprisingly, participants without aura scored lower than participants with aura. This study did have more migraine patients without aura participating than those with aura (21 with aura and 53 without aura), but they also noted one participant without aura undergo a migraine attack that presented with aura, suggesting either that the pathology of these two different types of migraines is not so far removed from each other, or that self-reporting migraine symptoms may be a somewhat unreliable test measure [29, 33].

Lastly, the most current case and eighth documented occurrence was reported in 2016 by Myren-Svelstad and colleagues. This case report details how a woman in her 40s was afflicted with a several-month-long migraine that progressed in intensity over time. Eventually, she also manifested the symptoms of prosopagnosia and topographic disorientation. MRI findings revealed a cerebral venous thrombosis in the right transverse sinus, which is located in the occipitotemporal region. This occipitotemporal region disruption and subsequent behavior fall in line with what is known about prosopagnosia pathology. Unfortunately, after the thrombosis was resolved, the patient still experienced low-grade migraine, implicating prolonged damage or dysfunction of this region [34].

## Conclusion and Future Directions

In summary, the case studies and recounts summarized here demonstrate that prosopagnosia can manifest as a symptom of migraine, particularly in patients experiencing aura. While unsurprising, this phenomenon is not frequently reported in the literature. These studies suggest a need for heightened clinical awareness of this potential overlap and suggest that further investigation into the cortical mechanisms underlying cases such as these could enhance our understanding of both migraine and prosopagnosia. Importantly, the consistent involvement of regions including the fusiform gyrus suggests a specific vulnerability in these networks during migraine attacks, particularly during the aura phase. Future research down this avenue should aim to quantify the prevalence of prosopagnosia, and similar symptoms, in migraine patients; for example, do prosopagnosic patients disproportionately experience migraine? If so, prosopagnosia occurring during migraine may serve as an indicator of more extensive cortical involvement, offering new insights into the broader, and perhaps longer term, neurological impacts of migraine.

## Key References

- Dai W, Liu RH, Qiu E, et al. (2021) Cortical mechanisms in migraine. Mol Pain 17:17448069211050246. 10.1177/17448069211050246.
  - ⚬ This reference provides in-depth coverage of cortical spreading depression, as well as the cortices’ proposed role in migraine pathology.
- Barton JJS. The 2024 Richardson Lecture: Prosopagnosia - A Classic Neurologic Deficit Meets the Modern Era. Can J Neurol Sci. 2024 Oct 11:1–9. doi: 10.1017/cjn.2024.295. Epub ahead of print. PMID: 39,391,940.
  - ⚬ This reference, written by an expert in the field, provides up to date information on current prosopagnosia diagnosis and pathology information.
- Hervias T (2024). An update on migraine: Current and new treatment options. JAAPA: official journal of the American Academy of Physician Assistants, 37(5), 1–7. 10.1097/01.JAA.0000000000000014.
  - ⚬ This reference provides updated information on migraine pathology, diagnosis, and treatment; written by an expert in the field.

## Data Availability

No datasets were generated or analysed during the current study.
